# Long-term oncological outcomes of transanal versus laparoscopic total mesorectal excision for mid-low rectal cancer: a retrospective analysis of 2502 patients

**DOI:** 10.1097/JS9.0000000000000992

**Published:** 2023-12-11

**Authors:** Ze Li, Huashan Liu, Shuangling Luo, Yujie Hou, Yebohao Zhou, Xiaobin Zheng, Xingwei Zhang, Liang Huang, Ziwei Zeng, Liang Kang

**Affiliations:** a Department of General Surgery (Colorectal Surgery); b Guangdong Provincial Key Laboratory of Colorectal and Pelvic Floor Diseases; c Biomedical Innovation Center, The Sixth Affiliated Hospital, Sun Yat-sen University, Guangzhou, China; d University Clinic Mannheim, Medical Faculty Mannheim, Heidelberg University, Mannheim, Germany

**Keywords:** laparoscopic surgery, local recurrence, rectal cancer, total mesorectal excision, transanal total mesorectal excision

## Abstract

**Background:**

Transanal total mesorectal resection (taTME) has recently emerged as a promising surgical approach for the treatment of mid-low rectal cancer. However, there is limited evidence on the long-term survival outcomes associated with taTME. This retrospective study aimed to compare the overall survival (OS), disease-free survival (DFS), and cancer-specific survival of taTME and laparoscopic TME (laTME) in patients with mid-low rectal cancer.

**Materials and Methods:**

From July 2014 to June 2022, a total of 3627 patients were identified from two prospective cohorts: the laparoscopic rectal surgery cohort and the CNTAES cohort. To balance the baseline characteristics between the taTME and laTME groups, propensity score matching (PSM) was performed.

**Results:**

A total of 2502 patients were included in the study. Prior to PSM, the laTME group comprised 1853 patients, while the taTME group comprised 649 patients. The 5-year OS (82.9% vs. 80.4%, *P*=0.202) and 5-year DFS (74.4% vs. 72.5%, *P*=0.167) were comparable between the taTME and laTME groups. After PSM, the taTME group showed no statistically significant difference in the 5-year OS (83.1% vs. 79.2%, *P*=0.101) and 5-year DFS (74.8% vs. 72.1%, *P*=0.135) compared to the laTME group. Subgroup analysis further suggested that taTME may potentially reduce the risk of death [hazard ratio 0.652; (95% CI, 0.452–0.939)] and disease recurrence [hazard ratio 0.736; (95% CI, 0.562–0.965)] specifically in patients with low rectal cancer.

**Conclusion:**

In this study, taTME demonstrated comparable oncologic safety to laTME in patients with mid-low rectal cancer. Moreover, the results indicate that taTME may confer potential survival benefits for patients with low rectal cancer.

## Introduction

Highlights

- In recent years, transanal total mesorectal resection (taTME) for mid-low rectal cancer has gained substantial attention due to its focus on oncological safety.
- This retrospective analysis leverages data from two prospective cohorts, spanning ~8 years and encompassing a total of 2502 patients.
- taTME demonstrates the potential to yield comparable long-term oncological outcomes to laparoscopic total mesorectal resection.
- The results of the subgroup analysis suggest that, for patients diagnosed with low rectal cancer, taTME may be considered a preferred surgical technique.

The management of rectal cancer inherently involves a multidisciplinary approach, with surgery playing a crucial role in the treatment process. Total mesorectal excision (TME) is the standard surgical procedure for mid-low rectal cancer^1^. With the rise of minimally invasive surgery, laparoscopic TME (laTME) has become increasingly popular^2^. Several randomized controlled trials (RCTs) have shown that laTME is comparable to open TME (opTME) in terms of oncological outcomes for mid-low rectal cancer^3–5^. In addition to achieving similar oncological outcomes, laTME has the advantage of enhancing recovery and reducing postoperative complications^6^. However, obese male patients with bulky tumours in a narrow pelvis pose a challenge for TME, regardless of the approach used. This is because transabdominal TME may result in suboptimal resected specimens, which can impact prognosis.

Transanal total mesorectal excision (taTME) is a novel technique that offers a bottom-to-top approach and may prove beneficial for treating challenging patients, particularly males with advanced lower rectal cancer^7–9^. Many studies have reported that taTME has the potential to produce better quality resected specimens and similar or better short-term outcomes. However, the long-term oncological results of taTME remain controversial. The Norwegian colorectal surgical community has postponed the use of taTME due to unacceptable early high local recurrence rates and rapid multifocal progression after the procedure^10^. While some retrospective studies have shown that the local recurrence rate of taTME is similar to that of laTME^11,12^, others suggest that a recurrence pattern similar to what was reported in Norway may occur in the early stages of the taTME learning curve^13^. These findings have raised concerns regarding the oncological outcomes of taTME.

As a relatively new technology introduced around a decade ago, it is essential to establish the safety and long-term oncological outcomes of taTME before it can be widely accepted. However, most medium and long-term outcome studies on taTME are based on single-arm studies with small sample sizes and without control groups, as evident in the existing literature^14–17^. Although the TaLaR trial has demonstrated the safe performance of taTME by experienced surgeons and its potential for earlier postoperative recovery^18^, it will take time to report long-term oncological results from this trial. Therefore, we conducted a retrospective study using data from two prospective cohorts to compare the long-term oncological outcomes between taTME and laTME.

## Method

### Study design

This retrospective analysis focused on rectal cancer patients treated at a single-centre. It has been registered at ClinicalTrial.gov. To ensure transparent and comprehensive reporting of our findings, we have adhered to the Strengthening the Reporting of Cohort Studies in Surgery (STROCCS) guidelines^19^, Supplemental Digital Content 1, http://links.lww.com/JS9/B519.

**Figure 1:**
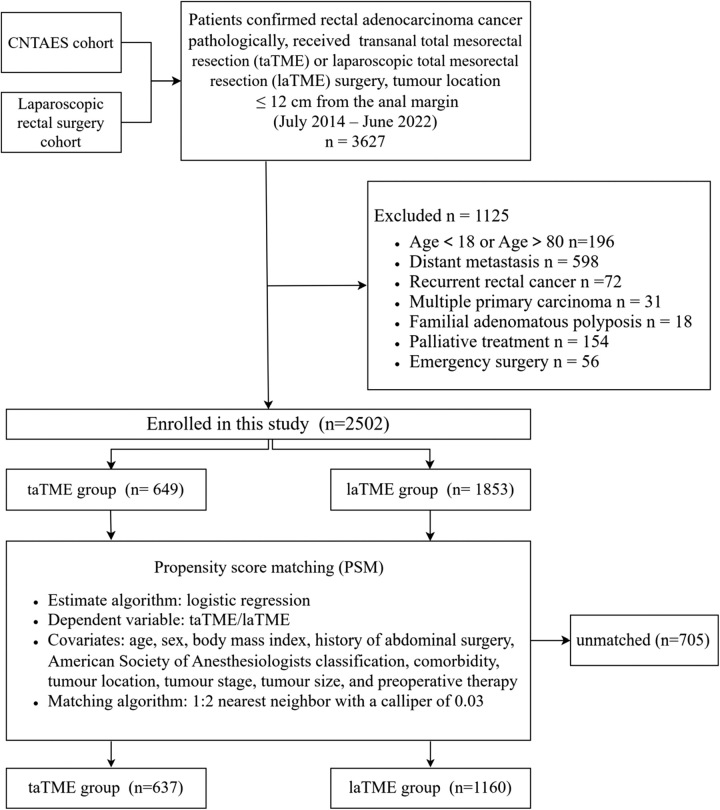
Flow diagram of patients included in the study.

We identified all patients between July 2014 and June 2022 from two prospective cohorts in our centre: the laparoscopic rectal surgery cohort and the CNTAES cohort. The CNTAES cohort comprises patients with rectal diseases who underwent transanal endoscopic surgery, such as local excision and taTME. We enroled patients with histologically confirmed rectal adenocarcinoma who underwent taTME or laTME surgery. The tumours were situated within 12 cm of the anal verge, as determined by MRI. We excluded patients who met any of the following criteria: (a) age over 80 or under 18 years, (b) emergency surgery, (c) palliative treatment, (d) distant metastasis, (e) multiple colorectal tumours at diagnosis, (f) recurrent rectal cancer, or (g) familial adenomatous polyposis. Figure 1 shows the screening process, and the enroled patients were divided into the taTME or laTME group.

### Outcome measurement

The primary outcome of the study was survival, including overall survival (OS), disease-free survival (DFS), and cancer-specific survival (CSS). The secondary outcome was local recurrence (LR). CSS was defined as the time from primary rectal cancer resection to death caused by rectal cancer. LR was defined as radiological or histopathological evidence of any recurrent disease deposit located in the pelvis within the area of dissection following primary rectal cancer resection, with or without distal metastasis. The location of local recurrence was classified using the system developed by Georgiou *et al*.^20^

The baseline clinical characteristics of patients included in the study were collected, including age, sex, body mass index (BMI), American Society of Anesthesiologists (ASA) classification, comorbidities, history of abdominal surgery, tumour distance, tumour size, clinical tumour stage, and preoperative treatment. The tumour distance and size data were obtained from preoperative computed tomography (CT) and MRI measurements. The tumour TNM stage was determined based on the 8th edition of the American Joint Committee on Cancer (AJCC) staging system^21^. The clinical stage and treatment plan for each patient were discussed by a multidisciplinary treatment (MDT) team. All patients receiving neoadjuvant therapy in this study were evaluated by experienced medical oncologists using either the FOLFOX or XELOX protocol for 4 to 6 cycles. Concurrent radiotherapy was delivered in 23–28 fractions over 5–6 weeks for a total dose of 46–50.4 Gy during chemotherapy cycles 2–4. Patients who underwent neoadjuvant radiotherapy had their surgery planned for 8–10 weeks after the last radiotherapy session.

In this study, the perioperative variables included the operative procedure, operative time, estimated blood loss, intraoperative blood transfusion, conversion to open surgery, type of anastomosis, enterostomy, intraoperative complications, and 30-day postoperative complications. The taTME procedure was performed by experienced surgeons in two groups^22^. The detailed surgical steps about taTME were available in the Supplementary Materials, Supplemental Digital Content 2, http://links.lww.com/JS9/B520. The decision to create a protective ileostomy depended on the reliability and blood supply of the anastomosis. Postoperative complications were graded according to the Clavien–Dindo classification^23^. Anastomotic leakage was diagnosed according to the definition of the International Study Group of Rectal Cancer^24^.

The histopathological variables included in the analysis were the distance between the tumour and the distal resection margin (DRM), number of lymph nodes harvested, positive rates of DRM (≤1 mm) and circumferential resection margin (CRM) (≤1 mm), differentiation grade, lymphovascular invasion, nerve invasion, and pathological tumour stage. The quality of resected mesorectum was assessed according to Quirke’s criteria^25^.

Follow-up data were obtained through outpatient or telephone consultations, following the recommended scheme outlined in the National Comprehensive Cancer Network (NCCN) guidelines. The research team cross-checked and verified all data to ensure accuracy, retrieving any missing or incomplete information from the electronic medical records system.

### Statistical methods

Statistical analyses were conducted using the Statistical Package for the Social Sciences Software (v26.0; SPSS Inc) and R (version 4.2.2, 2022-10-31). Continuous variables were reported as either mean ± standard deviation (interquartile range, IQR) or median (IQR), depending on their distribution, and were analyzed accordingly. Categorical variables were reported as the number of patients (percentage) and compared using the χ^2^ test or Fisher’s exact test, as appropriate. Statistical significance was set at *P* less than 0.05. Propensity score matching (PSM) analysis was performed using a 1:2 nearest neighbour matching algorithm to minimize confounding effects of age, sex, BMI, history of abdominal surgery, ASA Score, comorbidity, tumour location, tumour stage, tumour size, and preoperative therapy. The median follow-up time was calculated using the Reverse Kaplan–Meier method. Survival analyses were conducted using the Kaplan–Meier curve, and the log-rank test was used to compare survival curves. Univariate and multivariate Cox proportional hazard models were used to identify variables affecting OS and DFS. Variables with a *P* value less than 0.1 in the univariate analysis were included in the multivariate regression analysis. Hazard ratios (HR), 95% CI, and *P* values were used to interpret the results.

## Results

### Patient characteristics

As depicted in Fig. 1, we identified a total of 3627 patients with rectal cancer who received taTME or laTME surgery in our hospital from July 2014 to June 2022, from two prospective cohorts. Ultimately, we included 2502 patients in this study, with 649 patients in the taTME group and 1853 patients in the laTME group. To mitigate bias and ensure comparability between groups, we conducted PSM as detailed in the Methods. The baseline characteristics of patients before and after PSM are presented in Table 1. Before PSM, patients in the taTME group were found to be younger (56.5±12.5 vs. 58.0±11.7 years, *P*=0.031), with lower locations of tumours (50±18 vs. 64±27 mm, *P*<0.001), smaller tumour sizes (32±13 vs. 35±15 mm, *P*<0.001), and a higher likelihood of receiving neoadjuvant therapy (52.7% vs. 37.6%, *P*<0.001) when compared to the laTME group. Following PSM, we analyzed 637 patients in the taTME group and 1160 patients in the laTME group and found the baseline characteristics to be comparable between the two groups.

**Table 1:** Demographics and pretreatment tumour characteristics.

	Unmatched cohort			Matched cohort		
Characteristic	taTME (*n* = 649)	laTME (*n* =1853)	*P*	taTME (*n* = 637)	laTME (*n* =1160)	*P*
Age, mean ± SD (IQR), y	56.5±12.5 (49.0–66.0)	58.0±11.7 (51.0–67.0)	0.031	56.7±12.4 (49.0–66.0)	57.0±12.0 (50.0–66.0)	0.539
Sex			0.326			0.390
Male, *n* (%)	430 (66.3)	1188 (64.1)		422 (66.2)	745 (64.2)	
Female, *n* (%)	219 (33.7)	665 (35.9)		215 (33.8)	415 (35.8)	
BMI, mean ± SD (IQR)	22.9±3.2 (20.8–24.8)	22.7±3.0 (20.7–24.6)	0.560	22.9±3.2 (20.8–24.8)	22.7±3.0 (20.7–24.7)	0.128
Underweight or normal (<25.0), *n* (%)	498 (76.7)	1454 (78.5)		487 (76.5)	908 (78.3)	
Overweight (25.0–30.0), *n* (%)	138 (21.2)	371 (20.2)		137 (21.5)	236 (20.3)	
Obese (>30.0), *n* (%)	13 (2.0)	28 (1.5)		13 (2.0)	16 (1.3)	
History of abdominal surgery, *n* (%)	71 (10.9)	160 (8.6)	0.081	71 (11.1)	102 (8.8)	0.106
ASA Score, *n* (%)			0.500			0.291
I	360 (55.5)	1004 (54.2)		355 (55.7)	634 (54.7)	
II	267 (41.1)	799 (43.1)		260 (40.8)	499 (43.0)	
III	22 (3.4)	50 (2.7)		22 (3.5)	27 (2.3)	
Comorbidity			0.952			0.486
Yes	191 (29.4)	543 (29.3)		185 (29.0)	319 (27.5)	
No	458 (70.6)	1310 (70.7)		452 (71.0)	841 (72.5)	
Tumour distance from anal verge^a^, mean ± SD (IQR), mm	50±18 (36-61)	64±27 (43-83)	<0.001	50±19 (36-62)	51±21 (35-65)	0.191
51-100 mm, *n* (%)	262 (40.4)	969 (52.3)		262 (41.1)	512 (44.1)	
≤50 mm, *n* (%)	380 (58.6)	653 (35.2)		368 (57.8)	607 (52.3)	
Tumour size^a^, mean ± SD (IQR), mm	32±13 (24–38)	35±15 (24–42)	<0.001	32±13 (24–38)	32±13 (23–40)	0.942
Clinical TNM stage^a^, *n* (%)			0.301			0.412
I	83 (12.8)	253 (13.7)		80 (12.6)	172 (14.8)	
II	255 (39.3)	665 (35.9)		249 (39.1)	445 (38.4)	
III	311 (47.9)	935 (50.5)		308 (48.4)	543 (46.8)	
Preoperative therapy			<0.001			0.325
Chemoradiotherapy, *n* (%)	122 (18.8)	288 (15.5)		122 (19.2)	224 (19.3)	
Chemotherapy alone, n (%)	210 (33.9)	409 (22.1)		198 (31.1)	323 (27.8)	

ASA, American Society of Anesthesiologists; IQR, interquartile range; laTME, laparoscopic total mesorectal resection; taTME, transanal total mesorectal resection; TNM, tumour node metastasis.

a The data were extracted from preoperative computed tomography and MRI measurements.

### Perioperative outcomes and histopathological outcomes

Table S1, Supplemental Digital Content 2, http://links.lww.com/JS9/B520 and Table 2 presented the perioperative outcomes of the unmatched and matched cohorts. Regardless of whether the cohorts were matched, taTME procedures were associated with higher rates of sphincter preservation. It is worth noting that only one patient in the taTME group required conversion to open surgery, while 66 (3.6%) and 44 (2.9%) patients in the laTME group were converted before and after PSM, respectively. These differences were statistically significant (*P*<0.001). After PSM, the rate of enterostomy in the laTME group was significantly higher than in the taTME group (73.6% vs 46.3%, *P*<0.001). Before PSM, the complication rate was slightly lower in the taTME group compared to the laTME group, but the difference was not statistically significant (15.4% vs. 16.2%, *P*=0.663). However, after PSM, the complication rate was significantly higher in the laTME group compared to the taTME group (15.5% vs. 18.4%, *P*<0.001). There was no significant difference in the incidence of anastomotic leakage. In terms of histopathological outcomes, there was no significant difference between the two groups, both before and after PSM, including the quality of TME, DRM status, CRM status, number of harvested lymph nodes, lymphovascular invasion, nerve invasion, and tumour differentiation. In both unmatched and matched cohorts, the distance between the tumour and DRM was shorter in the taTME group than in the laTME group. Before PSM, the number of patients with pathological stage III in the laTME group was higher than in the taTME group (35.4% vs. 30.0%, *P*=0.048). However, after PSM, there was no significant difference in pathological staging between the two groups. The results of the correlational analysis are shown in Table S2, Supplemental Digital Content 2, http://links.lww.com/JS9/B520 and Table 3.

**Table 2:** Operative details and clinical outcomes of matched cohort.

	taTME (*n* = 637)	laTME (*n* =1160)	*P*
Operative procedure			
LAR	534 (83.8)	775 (66.8)	
ISR	98 (15.4)	185 (15.9)	<0.001
Hartmann	1 (0.2)	7 (0.6)	
APR	4 (0.6)	193 (16.6)	
Operative time mean ± SD (IQR), min	205.1±72.1 (155–239)	225.7±53.0 (196–244)	<0.001
Estimated blood loss, median (95% CI), ml	50 (73–121)	50 (93–110)	<0.001
Intraoperative blood transfusion, *n* (%)	9 (1.4)	35 (3.0)	0.038
Type of anastomosis, *n* (%)			<0.001
Stapled	445 (69.9)	745 (64.2)	
Handsewn	187 (29.4)	222 (19.1)	
Conversion to open surgery, *n* (%)	1 (0.2)	34 (2.9)	<0.001
Intraoperative complications, *n* (%)	7 (1.1)	25 (2.2)	0.135
Enterostomy, *n* (%)	295 (46.3)	854 (73.6)	<0.001
Diverting ileostomy	290 (45.5)	654 (56.4)	<0.001
Permanent or temporal colostomy	5 (0.8)	200 (17.2)	<0.001
30-d Postoperative complication^a^, *n* (%)	99 (15.5)	214 (18.4)	<0.001
Clavien–Dindo grade 3+ complications, *n* (%)	22 (3.4)	58 (5.0)	0.068
Anastomotic leak	63 (9.9)	93 (8.0)	0.189

APR, abdominal-perineal resection; IQR, interquartile range; ISR, intershipincteric resection; LAR, low anterior resection; laTME, laparoscopic total mesorectal resection; taTME, transanal total mesorectal resection.

a More than one complication could have occurred per patient.

**Table 3:** Histopathological outcomes of matched cohort.

	taTME (*n* = 637)	laTME (*n* =1160)	*P*
Quality of TME, n (%)			0.087
Complete	589 (92.5)	1044 (90.0)	
Nearly complete	48 (7.5)	116 (10.0)	
Length between tumour and DRM, mm, mean ± SD (IQR)	14±10 (6.5–18)	23±14 (10–30)	<0.001
Positive DRM, *n* (%)	3 (0.5)	10 (0.9)	0.266
Positive CRM, *n* (%)	4 (0.6)	11 (0.9)	0.594
Number of harvested lymph nodes, median, (IQR)	15 (11–21)	16 (11–21)	0.391
Lymphovascular invasion, *n* (%)	65 (10.2)	141 (12.2)	0.246
Nerve invasion, *n* (%)	69 (10.8)	142 (12.2)	0.400
Pathology stage, *n* (%)			0.128
0 + PCR	53 (8.3)	123 (10.6)	
I	187 (29.4)	304 (27.7)	
II	203 (31.9)	324 (28.2)	
III	194 (30.5)	409 (33.5)	
Tumour differentiation, *n* (%)			0.140
Well	59 (9.2)	138 (11.9)	
Moderate	509 (79.9)	871 (75.1)	
Poor	19 (3.0)	43 (3.7)	
PCR	50 (7.8)	108 (9.3)	

CRM, circumferential resection margin; DRM, distal resection margin; IQR, interquartile range; laTME, laparoscopic total mesorectal resection; PCR, pathologic complete response; taTME, transanal total mesorectal resectionTME, Total mesorectal resection.

### Long-term oncological outcomes

**Figure 2:**
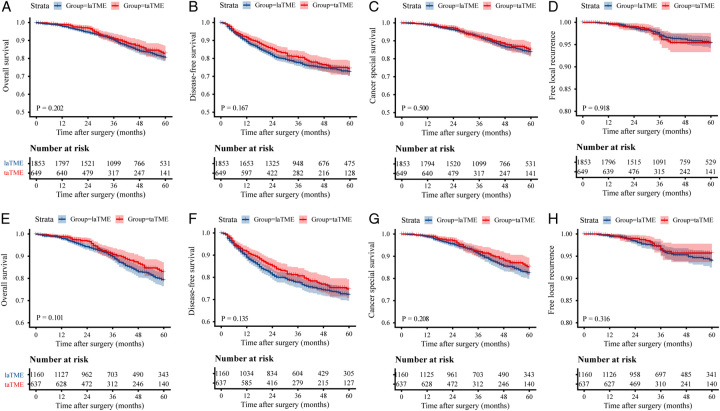
Kaplan–Meier curves of OS (A), DFS (B), CSS (C) and free-LR (D) in the unmatched cohort. Kaplan–Meier curves of OS (E), DFS (F), CSS (G) and free-LR (H) in the matched cohort. CSS, cancer-specific survival; DFS, disease-free survival; laTME, laparoscopic total mesorectal resection; LR, local recurrence; OS, overall survival; taTME, transanal total mesorectal resection.

The patients in the taTME group were followed up for a median time of 38 months, while those in the laTME group were 44 months. The long-term oncology results of both groups, both before and after PSM, are presented in Table S3, Supplemental Digital Content 2, http://links.lww.com/JS9/B520. Kaplan–Meier curves of OS, DFS, CSS, and LR of the unmatched and matched groups are depicted in Fig. 2.

The taTME and laTME groups showed similar estimated 3-year OS rates (90.9% vs. 90.4%, *P*=0.434) and 3-year DFS rates (80.6% vs. 77.7%, *P*=0.092) before PSM, and these estimates remained similar after PSM. In the unmatched cohort, the taTME group had a 5-year OS rate of 82.9% (95% CI, 78.8–87.0%), which was similar to the 80.4% (95% CI, 78.0–82.8%) rate in the laTME group. In the matched cohort, no statistically significant difference was observed between taTME and laTME groups in 5-year OS rate (83.1% vs. 79.2%, *P*=0.101). Regarding the 5-year DFS, there were no significant differences between the two groups before and after PSM (74.4% vs. 72.5%, *P*=0.167; 74.8% vs. 72.1%, *P*=0.135).

Before PSM, the cumulative 3-year and 5-year LR rates were similar between the taTME group [3.2% (95% CI, 1.4–5.0%) and 4.6% (95% CI, 2.4–6.8%), respectively] and the laTME group [2.8% (95% CI, 2.0–3.6%) and 4.7% (95% CI, 3.3–6.1%), respectively]. In the matched cohort, there was no statistically significant difference in the cumulative 3-year and 5-year LR rates bewteen the taTME group [3.3% (95% CI, 1.5–5.1%) and 4.3% (95% CI, 2.1–6.5%), respectively] and the laTME group [3.6% (95% CI, 2.4–4.8%) and 6.1% (95% CI, 4.3–7.9%), respectively]. Table S4, Supplemental Digital Content 2, http://links.lww.com/JS9/B520 presents the distribution of local recurrence sites, where the central anastomotic area is identified as the primary site. Interestingly, a subset of our cohort demonstrated local recurrence after undergoing surgery during the early phase of the taTME learning curve. This finding aligns with the results reported in our previous study, which specifically addressed this topic^26^.

### Cox proportional hazards regression analysis and subgroup analysis

The Cox Proportional Hazards Regression Analysis results are presented in Table 4. Multivariate Cox regression analysis demonstrated that the surgical technique did not have an independent association with OS (HR 0.880, 95% CI 0.674–1.169). However, several factors were identified to be significantly associated with an unfavourable survival outcome. These factors included ASA greater than or equal to 3 (indicating severe physical status), tumour distance from the dentate line less than or equal to 50 mm, tumour size, cT4 tumour stage, preoperative therapy, pT4 tumour stage, pN+ tumour stage, nerve invasion, lymphovascular invasion, and increased age. Additionally, no relationship between the surgical technique and disease-free survival (DFS) was observed based on the Cox regression analysis (HR 0.873, 95% CI 0.713–1.069).

**Table 4:** Multivariate cox proportional hazards regression analysis.

	Overall survival			Disease-free survival		
	HR	95% CI	*P*	HR	95% CI	*P*
Approach taTME	0.888	0.674–1.169	0.396	0.873	0.713–1.069	0.189
Age	1.017	1.007–1.027	0.001	1.006	0.999–1.013	0.091
Female	0.798	0.625–1.019	0.071	0.810	0.674–0.973	0.025
High ASA Grade (≥ 3)	2.210	1.358–3.598	0.001	1.807	1.217–2.684	0.003
Obesity (BMI ≥25)	0.961	0.729–1.265	0.775	0.975	0.794–1.198	0.812
Tumour distance from anal verge ≤50 mm	1.519	1.211–1.905	<0.001	1.285	1.081–1.529	0.005
Tumour size	1.117	1.041–1.199	0.002	1.099	1.041–1.160	0.001
cT4	1.492	1.081–2.060	0.015	1.384	1.086–1.764	0.009
cN+	1.040	0.820–1.319	0.748	1.012	0.844–1.212	0.899
Preoperative therapy	1.912	1.512–2.417	<0.001	1.916	1.603–2.290	<0.001
pT4	1.769	1.186–2.637	0.005	1.536	1.112–2.121	0.009
pN+	1.925	1.498–2.473	<0.001	2.128	1.764–2.569	<0.001
PCR	0.507	0.272–0.945	0.032	0.480	0.299–0.769	0.002
Nerve invasion	2.125	1.592–2.835	<0.001	1.612	1.293–2.008	<0.001
Lymphovascular invasion	1.615	1.202–2.169	0.001	1.501	1.198–1.881	<0.001

ASA, American Society of Anesthesiologists; HR, hazard ratio; N, lymph node; PCR, pathologic complete response; T, tumour; taTME, transanal total mesorectal excision.

The survival subgroup analyses are presented in Figure S1, Supplemental Digital Content 2, http://links.lww.com/JS9/B520 and S2, Supplemental Digital Content 2, http://links.lww.com/JS9/B520. The analysis of subgroups based on gender, obesity, and Chemoradiotherapy did not reveal any significant differences between the two surgical methods in terms of both OS and DFS. Improved overall and disease-free survival rates were observed in patients with a tumour distance from the dentate line ≤50 mm who underwent taTME compared to those who underwent laTME [HR=0.652, (95% CI, 0.452–0.939) and HR=0.736, (95% CI, 0.562–0.965), respectively]. These findings suggest that taTME may have a beneficial impact on long-term outcomes in this specific patient population.

## Discussion

In recent years, concerns about the oncological safety of taTME have been raised worldwide, largely due to its suspension in Norway. Several large RCTs comparing taTME and laTME are currently underway, but only the TaLaR trial has reported short-term results^18^. To address this research gap, we conducted a study based on two prospective cohorts to analyze the long-term oncological outcomes of taTME and laTME. Our study findings revealed that taTME was associated with a lower rate of protective ileostomy and faster postoperative recovery in the short term. Importantly, we found that the oncological outcomes were similar between the two surgical approaches. Moreover, after PSM and subgroup analysis, our results suggested that taTME might yield better oncological outcomes specifically for patients with low rectal cancer.

The TaLaR trial and meta-analyses have demonstrated the reliable perioperative safety of taTME and its superior postoperative recovery compared to laTME^18,27^. Consistent with these findings, our study also found similar perioperative results between the two surgical methods. However, in the matched cohort analysis, the taTME group had a significantly lower postoperative complication rate compared to the laTME group (15.5% vs 18.4%, *P*<0.001). As the proportion of patients with low rectal cancer increased after PSM, this difference may reflect the unique advantage of transanal surgery over laparoscopic surgery in patients with low rectal cancer. Additionally, the quality of surgery can be assessed based on indicators such as complete or nearly complete TME, clear (>1 mm) CRM, and clear (>1 mm) DRM^28,29^. These indicators are crucial in determining the patient’s prognosis^30^. Although none of these three indicators showed significant differences between the two groups in our study, the taTME group had a higher proportion of patients with complete TME resection and fewer patients with positive CRM and DRM. This suggests a promising outlook for taTME in terms of surgical quality.

While taTME has demonstrated comparable surgical quality to laTME, confirming its long-term oncological outcomes is essential. Previous single taTME cohort studies by Maykel *et al.* ^15^ and Sanchon *et al.* ^31^ reported 3-year OS and 3-year DFS of 86.6% and 82.6%, respectively, and 5-year OS and 5-year CSS of 76.6% and 87%, respectively. The International TaTME Registry Collaborative, which included 2803 patients with a median follow-up of 2 years, reported 2-year DFS and OS rates of 77% (95% CI, 75–79%) and 92% (95% CI, 91–93%), respectively^32^. A multicenter cohort study by Burghgraef *et al.* ^33^ also found no significant difference in long-term oncological outcomes between taTME and laTME, and multivariate Cox regression analysis did not show any influence of the surgical approach on survival. In our study, although not statistically significant, the 3-year and 5-year OS/DFS/CSS rates were slightly higher in the taTME group compared to the laTME group, both in unmatched and matched cohorts. To ensure surgical quality, abdominal-perineal resection (APR) is preferred for low rectal cancer to facilitate the most challenging part of the procedure. However, if APR is performed, patients must have a permanent colostomy, which is unacceptable to many patients. Studies comparing taTME and APR in the treatment of low rectal cancer have reported similar long-term effects^34^. RCTs have shown that transanal perineal dissection can reduce the risk of CRM involvement by approximately four-fold compared to abdominal TME in patients with low rectal cancer^35^. In our subgroup analysis of low rectal tumours, taTME was associated with about 30% reduction in the risk of death, disease recurrence, and metastasis, suggesting that taTME might be the preferred technique for patients with low rectal cancer. However, further investigation is needed to confirm the long-term benefits of taTME.

Various factors can influence the long-term oncological outcomes in rectal cancer surgery, including tumour stage and biological behaviour. However, the quality of surgical technique plays a crucial role as it can directly impact LR rates. This study involves a comparison of LR rates between taTME and laTME procedures. The median follow-up time for both groups was over three years. After employing PSM, the local cumulative recurrence rate of taTME at 3 years was 3.3%, slightly lower than the 3.6% observed in the laTME group, although the difference was not statistically significant. Notably, a previous report from the Norwegian Colorectal Cancer Group highlighted an alarmingly high estimated local recurrence rate of 11.6% at 2.4 years after taTME surgery^36^. This high rate in Norwegian patients can likely be attributed to deficiencies in the execution of surgical procedures rather than inherent technological shortcomings. However, it is worth mentioning that the 3-year local recurrence rate in our study aligns with the long-term outcomes reported in other trials, such as COLOR II, ALaCART, and ACOSOG Z6051, which all reported rates around 5% for laTME^3,28,29^. Additionally, Lacy *et al*.^37^ demonstrated a potential reduction in the risk of local recurrence by 60% in stage II-III rectal cancer through taTME, with recurrence rates of 3.6% and 9.6% in the two groups (*P*=0.001). However, our study did not replicate these findings. Further analysis revealed that the eight relapses observed in the taTME group were conducted by four surgeons during the early learning curve phase. Nonetheless, after undergoing qualified structured training and successfully navigating the learning curve, taTME could potentially deliver reliable oncologic safety.

This study possesses certain limitations that should be acknowledged. Firstly, the retrospective nature of this single-centre cohort study introduces the possibility of biases, despite the meticulous data collection from a prospectively maintained and externally audited database. To minimize selection bias and enhance external validity, we employed PSM and multivariate analysis techniques. Secondly, we did not assess functional and quality of life outcomes, which are also crucial considerations in clinical decision-making. Although some studies and meta-analyses have shown no significant differences in long-term postoperative anal function between taTME and laTME^38^, it remains unclear whether the required anastomotic level and transanal platform for taTME could significantly impact anal function and quality of life. Further RCTs are warranted to evaluate this aspect thoroughly.

## Conclusion

Our study provides robust evidence supporting the feasibility and oncologic safety of taTME in proficient surgical centres. It has been observed that taTME exhibits comparable oncologic outcomes to the conventional laTME method. Remarkably, taTME presents a potential advantage in enhancing survival outcomes, specifically among patients with low rectal cancer. These findings advocate for the adoption of taTME as a management approach for middle and low rectal cancer, particularly when performed by skilled practitioners proficient in this technique. Nevertheless, continuous long-term monitoring is crucial to confirm the enduring effectiveness and safety of this novel surgical procedure.

## Ethical approval

This study involved human participants and approved by the Institutional Review Board of Sun Yat-sen University. The reference number is 2023ZSLYEC- 041. This study complies with the Declaration of Helsinki. All the participated patients signed informed consent.

## Consent

Written informed consent was obtained from the patient for publication of this case report and accompanying images. A copy of the written consent is available for review by the Editor-in-Chief of this journal on request.

## Source of funding

This work is supported by Science and Technology Projects in Guangzhou (202206010062), China Postdoctoral Science Foundation (2021M703723), Guangdong Basic and Applied Basic Research Foundation (2021A1515111011, and 2022A1515012498), National Natural Science Foundation of China (82000515), Sun Yat-sen University Clinical Research 5010 Program (2016005), Shenzhen “San Ming Projects” Research (Grant No.lc202002) and National Key Clinical Discipline. The funding is Guangdong Provincial Clinical Research Center for Digestive Diseases (2020B1111170004).

## Author contribution

Z.L., H.L., and S.L.: conceptualization, data curation, methodology, validation, visualization, and writing-original draft. Y.Z., Y.H., X.Z., and X.Z.: validation, formal analysis, resources, writing-review and editing. L.H., Z.Z. and L.K.: conceptualization, writing-original draft, writing-review and editing, funding acquisition and supervision. All authors read and approved the final manuscript.

## Conflicts of interest disclosure

The authors declare that they have no conflict of interest.

## Research registration unique identifying number (UIN)

1. Name of the registry: ClinicalTrials.gov.
2. Unique Identifying number or registration ID: NCT05682794.
3. Hyperlink to your specific registration (must be publicly accessible and will be checked): https://clinicaltrials.gov/ct2/show/NCT05682794?term=NCT05682794&draw=2&rank=1.

## Guarantor

Liang Kang.

## Data statement

The data derived from electronic medical record system in The Sixth Affiliated Hospital, Sun Yat-sen University. Due to the sensitive nature of the data in this study, information and data of the patients were assured raw data would remain confidential and would not be shared.

## Supplementary Material

SUPPLEMENTARY MATERIAL
