# Supplementary material for: Long-term oncological outcomes of transanal versus laparoscopic total mesorectal excision for mid-low rectal cancer: a retrospective analysis of 2502 patients
Source: Int J Surg. 2023 Dec 11;110(3):1611–9. doi: 10.1097/JS9.0000000000000992 (PMC10942237; doi:10.1097/JS9.0000000000000992)
Supplement: SUPPLEMENTARY MATERIAL [file js9-110-1611-s002.docx]

**Title: Long-term Oncological Outcomes of Transanal Versus Laparoscopic Total Mesorectal Excision for Mid-Low Rectal Cancer: A Retrospective Analysis of 2502 Patients in an Experienced Center**

**Authors:**

^*^Ze Li^1,2,3^ MD, ^*^Huashan Liu^1,2,3^ MD, PhD, ^*^Shuangling Luo^1,2,3^, MD, PhD, Yujie Hou^1,2,3^MD, Yebohao Zhou^1,2,3^MD, Xiaobin Zheng^1,2,3^MD Xingwei Zhang^1,2,3^MD, ^#^Liang Huang^1,2,3^MD, PhD, ^#^Ziwei Zeng^1,2,3,4^MD, and ^#^Liang Kang^1,2,3^MD, PhD

**Affiliations:**

1.Department of General Surgery (Colorectal Surgery), The Sixth Affiliated Hospital, Sun Yat-sen University

2. Guangdong Provincial Key Laboratory of Colorectal and Pelvic Floor Diseases, The Sixth Affiliated Hospital, Sun Yat-sen University

3. Biomedical Innovation Center, The Sixth Affiliated Hospital, Sun Yat-sen University

4.University Clinic Mannheim, Medical Faculty Mannheim, Heidelberg University, Mannheim, Germany

^*^These authors contributed equally.

^#^**Correspondence:**

Liang Huang, MD, PhD

Department of Colorectal Surgery,

Guangdong Institute of Gastroenterology,

Guangdong Provincial Key Laboratory of Colorectal and Pelvic Floor Diseases,

The Sixth Affiliated Hospital of Sun Yat-sen University, Guangzhou, Guangdong 510655, P. R. China.

Email: huangl75@mail.sysu.edu.cn

Ziwei Zeng, MD

Department of Colorectal Surgery,

Guangdong Institute of Gastroenterology,

Guangdong Provincial Key Laboratory of Colorectal and Pelvic Floor Diseases,

The Sixth Affiliated Hospital of Sun Yat-sen University, Guangzhou, Guangdong 510655, P. R. China.

University Clinic Mannheim, Medical Faculty Mannheim, Heidelberg University, Mannheim, Germany

Email. zengzw@mail2.sysu.edu.cn

Liang Kang, MD, PhD

Department of Colorectal Surgery,

Guangdong Institute of Gastroenterology,

Guangdong Provincial Key Laboratory of Colorectal and Pelvic Floor Diseases,

The Sixth Affiliated Hospital of Sun Yat-sen University, Guangzhou, Guangdong 510655, P. R. China.

Email. kangl@mail.sysu.edu.cn

**Supplementary Materials - Index**

| **Supplementary Tables and Figure** |  |
| --- | --- |
| Table S1 | *pag. 4* |
| Table S2 | *pag. 5* |
| Table S3 | *pag. 6* |
| Table S4 | *pag. 7* |
| Figure S1 | *pag. 8* |
| Figure S2 | *pag. 9* |
| Supplementary Text | *pag. 10* |

**Supplementary Tables**

Table S1. Operative details and clinical outcomes of the unmatched cohort

|  | taTME (n = 649) | laTME (n =1853) | P value |
| --- | --- | --- | --- |
| Operative procedure |  |  | <0.001 |
| LAR | 536(82.6) | 1435(77.4) |  |
| ISR | 108(16.6) | 195(10.5) |  |
| Hartmann | 1(0.2) | 16(0.9) |  |
| APR | 4(0.6) | 207(11.2) |  |
| Operative time mean ± SD (IQR), min | 204.7±72.4  (155-237) | 221.2±49.4  (195-241) | <0.001 |
| Estimated blood loss,median (95%CI), mL | 50  (75-123) | 50  (88-99) | <0.001 |
| Intraoperative blood transfusion, n (%) | 10(1.5) | 50(2.7) | 0.103 |
| Type of anastomosis, n (%) |  |  | <0.001 |
| Stapled | 448(69.0) | 1400(75.6) |  |
| Handsewn | 196(30.2) | 246(13.3) |  |
| Conversion to open surgery, n (%) | 1(0.2) | 66(3.6) | <0.001 |
| Intraoperative complications, n (%) | 8(1.2) | 35(1.9) | 0.298 |
| Enterostomy, n (%) | 301(46.4) | 1119(60.4) | <0.001 |
| Diverting ileostomy | 296(45.6) | 896(48.4) | 0.228 |
| Permanent or temporal colostomy | 5(0.8) | 223(12.1) | <0.001 |
| 30-d Postoperative complication^a^, n (%) | 100(15.4) | 301(16.2) | 0.663 |
| Clavien-Dindo grade 3+ complications, n (%) | 23(3.5) | 79(4.2) | 0.489 |
| Anastomotic leak | 64(9.8) | 139(7.5) | 0.058 |

1. More than one complication could have occurred per patient

*LAR* Low anterior resection，*ISR* Intershipincteric resection, *APR* Abdominal-perineal resection, *SD* Standard deviation, *IQR* Interquartile range, *CI* Confidence intervals.

Table S2. Histopathological outcomes of the unmatched cohort

|  | taTME (n = 649) | laTME (n =1853) | P value |
| --- | --- | --- | --- |
| Quality of TME, n (%) |  |  | 0.371 |
| Complete | 599(92.3) | 1688(91.1) |  |
| Nearly complete | 50(7.7) | 165(8.9) |  |
| Length between tumor and DRM, mm, mean ± SD (IQR) | 14±11  (6.5-17.5) | 26±15  (15-35) | <0.001 |
| Positive DRM, n (%) | 3(0.5) | 13(0.7) | 0.775 |
| Positive DRM, n (%) | 4(0.6) | 15(0.8) | 0.795 |
| Number of harvested lymph nodes, median, (IQR) | 15(11-21) | 17(12-23) | <0.001 |
| Lymphovascular invasion, n (%) | 65(10.0) | 243(13.1) | 0.440 |
| Nerve invasion, n (%) | 69(10.6) | 247(13.3) | 0.860 |
| Pathology stage, n (%) |  |  | 0.048 |
| 0 + PCR | 54(8.3) | 167(9.0) |  |
| I | 192(29.6) | 473(25.5) |  |
| II | 208(32.0) | 557(30.1) |  |
| III | 195(30.0) | 690(35.4) |  |
| Tumor differentiation, n (%) |  |  | 0.178 |
| Well | 61(9.4) | 234(12.6) |  |
| Moderate | 518(79.8) | 1429(77.1) |  |
| Poor | 19(2.9) | 55(3.0) |  |
| PCR | 51(7.9) | 135(7.3) |  |

*TME* Total mesorectal resection, *DRM* Distal resection margin, *SD* Standard deviation, *IQR* Interquartile range, *CRM* Circumferential resection margin, *PCR* Pathologic complete response.

Table S3. Oncology outcomes

*IQR* Interquartile range, *CI* Confidence intervals, *OS* Overall survival, *DFS* Disease-free survival, *CSS* Cancer-specific survival, *LR* Local recurrence.

|  | Before PSM | | | After PSM | | |
| --- | --- | --- | --- | --- | --- | --- |
|  | taTME | laTME | *P* value | taTME | laTME | *P* value |
| Median follow-up: months (IQR) | 38  (23-55) | 44  (27-61) |  | 38  (23-55) | 48  (27-62) |  |
| 3-Year OS rate in percent (95% CI) | 90.9%  (88.2%-93.6%) | 90.4%  (89.0%-91.8%) | 0.434 | 90.8%  (88.1%-93.5%) | 89.8%  (87.8%-91.8%) | 0.336 |
| 3-Year DFS rate in percent (95% CI) | 80.6%  (77.3%-83.9%) | 77.7%  (75.7%-79.7%) | 0.092 | 80.7%  (77.4%-84.0%) | 77.9%  (75.4%-80.4%) | 0.110 |
| 3-Year CSS rate in percent (95% CI) | 92.2%  (89.7%-94.7%) | 92.2%  (90.8%-93.6%) | 0.856 | 92.1%  (89.6%-94.6%) | 91.2%  (89.4%-93.0%) | 0.479 |
| 3-Year cumulative LR rate in percent (95% CI) | 3.2%  (1.4%-5.0%) | 2.8%  (2.0%-3.6%) | 0.773 | 3.3%  (1.5%-5.1%) | 3.6%  (2.4%-4.8%) | 0.660 |
| 5-Year OS rate in percent (95% CI) | 82.9%  (78.8%-87.0%) | 80.4%  (78.0%-82.8%) | 0.202 | 83.1%  (79.0%-87.2%) | 79.2%  (76.3%-82.1%) | 0.101 |
| 1. Year DFS rate in percent (95% CI) | 74.4%  (70.1%-78.7%) | 72.5%  (70.1%-74.9%) | 0.167 | 74.8%  (70.5%-79.1%) | 72.1%  (69.2%-75.0%) | 0.135 |
| 5-Year CSS rate in percent (95% CI) | 85.2%  (92.7%-83.7%) | 83.7%  (88.1%-83.3%) | 0.500 | 85.4%  (81.5%-89.3%) | 82.4%  (79.5%-85.3%) | 0.208 |
| 5-Year cumulative LR rate in percent (95% CI) | 4.6%  (2.4%-6.8%) | 4.7%  (3.3%-6.1%) | 0.865 | 4.3%  (2.1%-6.5%) | 6.1%  (4.3%-7.9%) | 0.362 |

Table S4. Feature of local recurrence

|  | taTME  n=20 | laTME  n=64 |
| --- | --- | --- |
| Location of local recurrence, n (%) |  |  |
| Anterior | 1(5.0) | 4(6.2) |
| Posterior | 3(15.0) | 10(15.6) |
| Lateral | 0 | 6(9.3) |
| Inferior | 0 | 5(7.8) |
| Central anastomotic | 11(55.0) | 22(34.4) |
| Central non-anastomotic | 3(15.0) | 11(17.1) |
| Others | 2(10.0) | 6(9.3) |
| Time to LR in months, median (range) | 14.5(5-40) | 15(1-47) |
| LR Patients received surgery, n (%) | 7(35.0) | 15(23.4) |
| LR Patients with pathologically proven adenocarcinoma, n (%) | 13(65.0) | 21(32.8) |
| Surgeon in the early learning curve  (The first 42 patients), n (%) | 8(40.0)^*^ |  |

* These patients’ surgery was undergone by four surgeons who during the early stage of learning curve at that time.

*LR* Local recurrence

**Supplementary Figures**

Figure S1. Subgroup analysis of overall survival


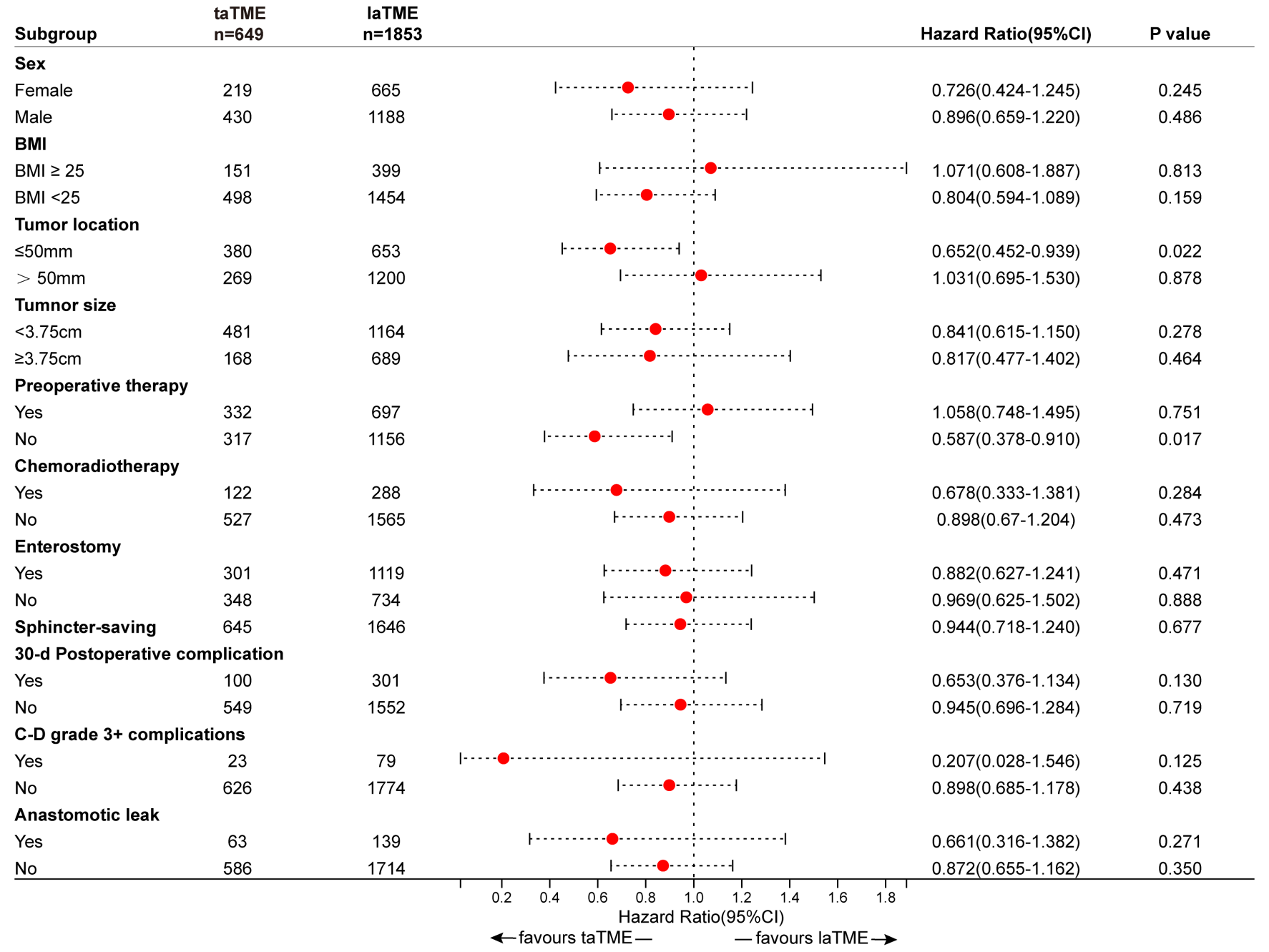


*BMI* Body mass index, *C-D* Clavien-Dindo.

Figure S2. Subgroup analysis of disease-free survival


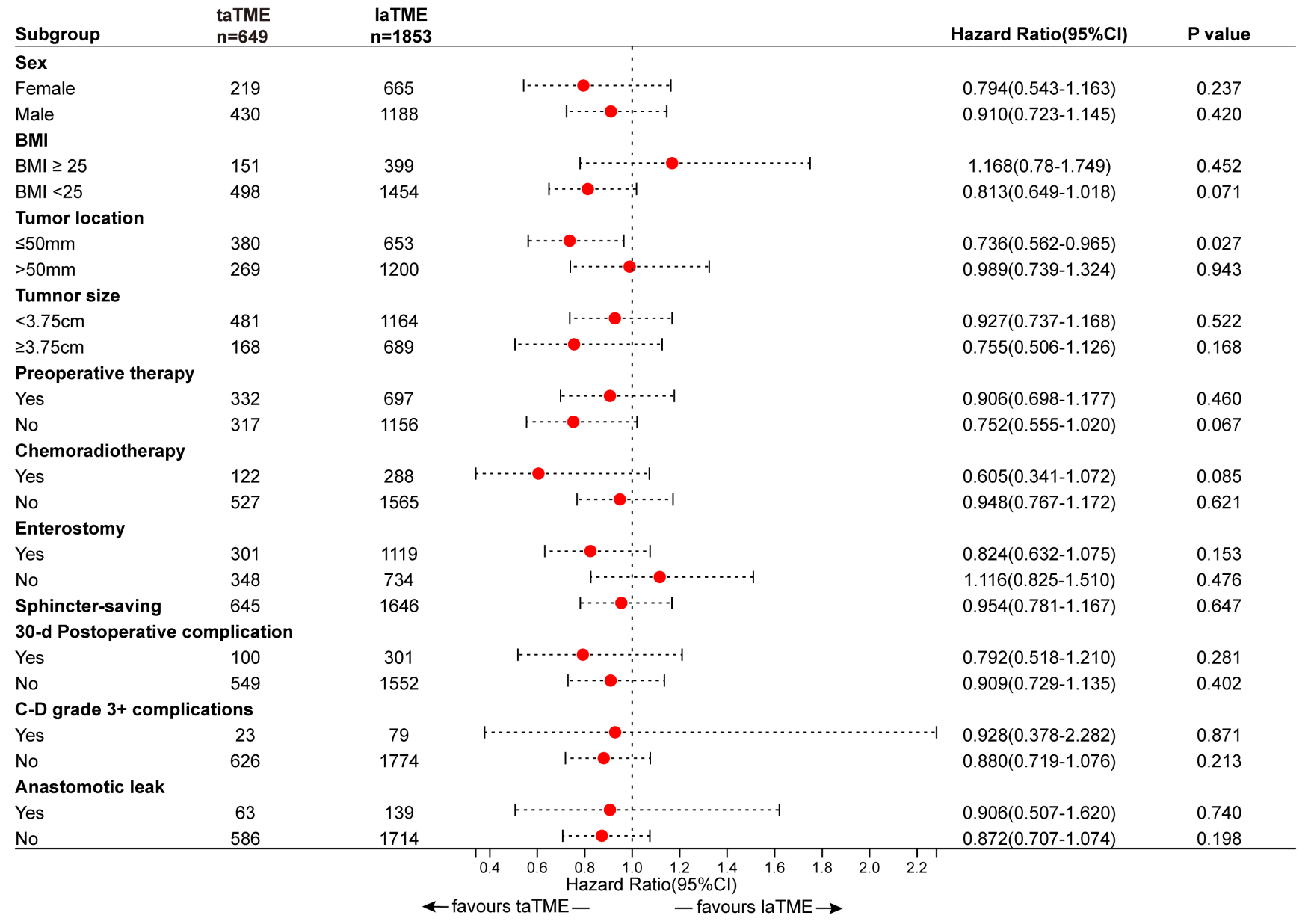


*BMI* Body mass index, *C-D* Clavien-Dindo.

Supplementary Text

**Transanal total mesorectal resection (taTME) surgical steps**

The technical approach of the pure or hybrid taTME was previously described^[1–3]^. Regardless of its sequence, the surgical procedure can be mainly divided into two groups: the trans-abdominal approach and the taTME.

The patients were placed in an extended lithotomy position. After abdominal and perineal disinfection, two groups of surgeons operated transabdominal and transanal, respectively and simultaneously.

Goals of the transabdominal procedure included separation of the inferior mesenteric vessels (IMV), mobilization of the left colon (the splenic flexure was mobilized as needed), and total mesorectal excision to the level of peritoneal reflection in most cases. All cases were routinely ligated at the root of the IMV.

After digital anal dilation, the transanal surgeon group used Lone star® retractor system (Hangtiankadi, Beijing, China) to sufficiently expose the anorectum to display the tumor. The taTME portion involved the following key steps: (i) utilization of the purse string to occlude the rectal lumen; (ii) full-thickness dissection of the rectal wall and perirectal fat until the mesorectal plane was identified; (iii) setting-up of the transanal multi-channel working platform and establishing the pneumo-anorectum by insufflating CO2; (iv) carefully approaching the “Holy plane” identified in step 2 circumferentially and cephalically until the peritoneal cavity was entered; If an intersphincteric resection was required for a tumor located <5cm from the anal verge, the sequence of steps 1 and 2 might be switched. For a middle rectal tumor, step 3 was performed prior to steps 1 and 2. During step 4, attention should be paid not to injure the vagina, urethra, presacral vessel, or the autonomic and parasympathetic nerve plexus.

The specimen was extracted either transabdominally or transanally with a preference for the hybrid taTME when the tumor was not too bulky. End-to-end stapled anastomosis with a diameter of 29mm was routinely constructed. Hand-sewn sutures were added for reinforcement if considered necessary. No end-to-side anastomoses, coloplasty, or colonic J-pouch was employed. Anastomoses test mainly depended on direct visualization of the donuts and blood supply by surgeons.

Below are pictures of the main surgical steps.

**Pictures of the main surgical steps**


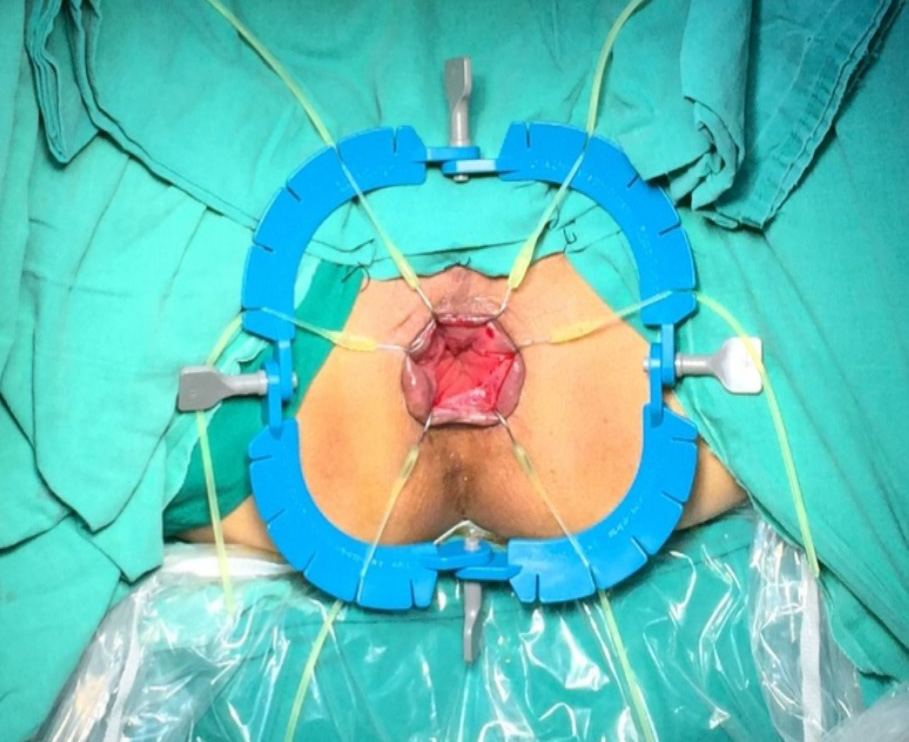


The transanal surgeon group used Lone star® retractor system (Hangtiankadi, Beijing, China) to sufficiently expose the anorectum to display the tumor.


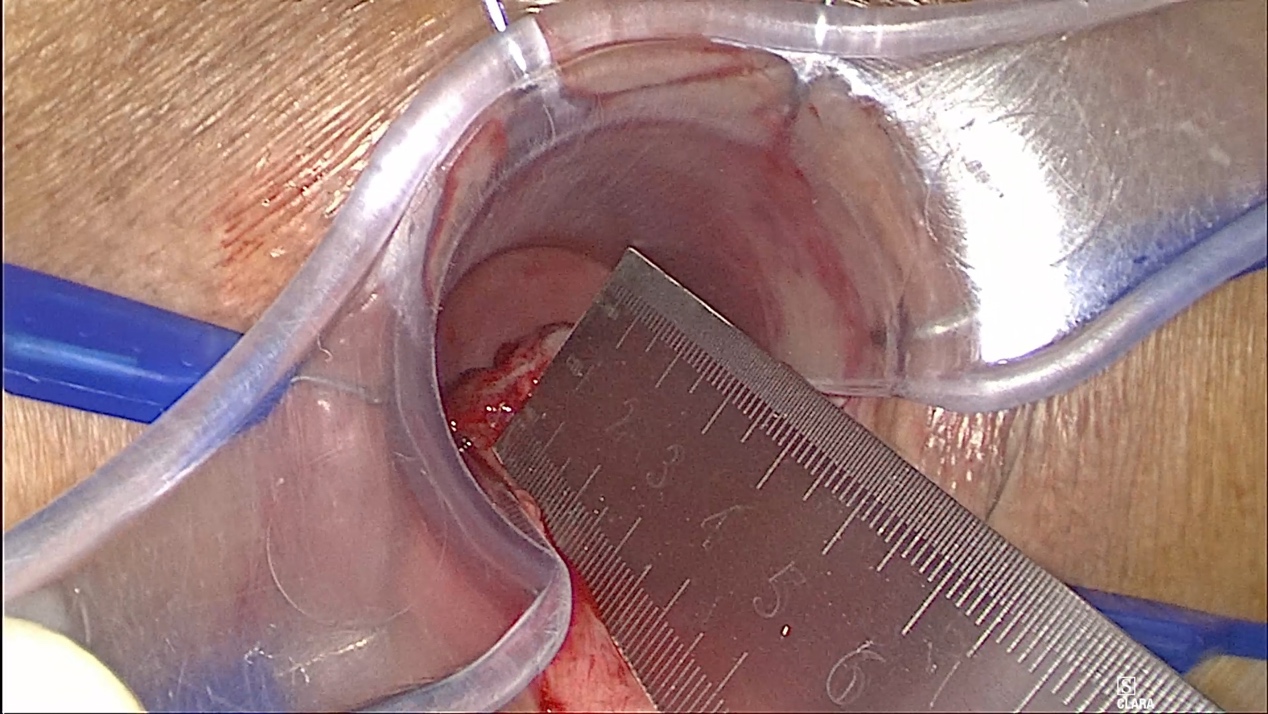


Semicircular anal speculum to expose the tumor


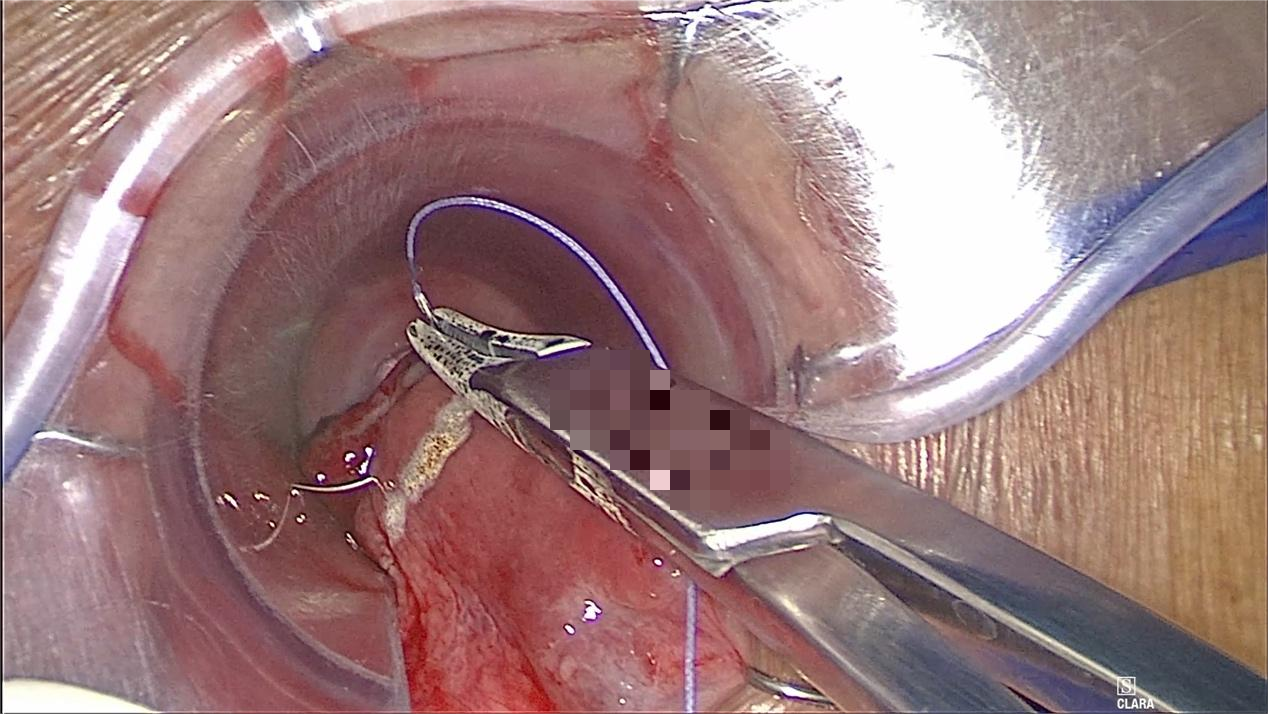


1. utilization of the purse string to occlude the rectal lumen and wash the lavage lumen


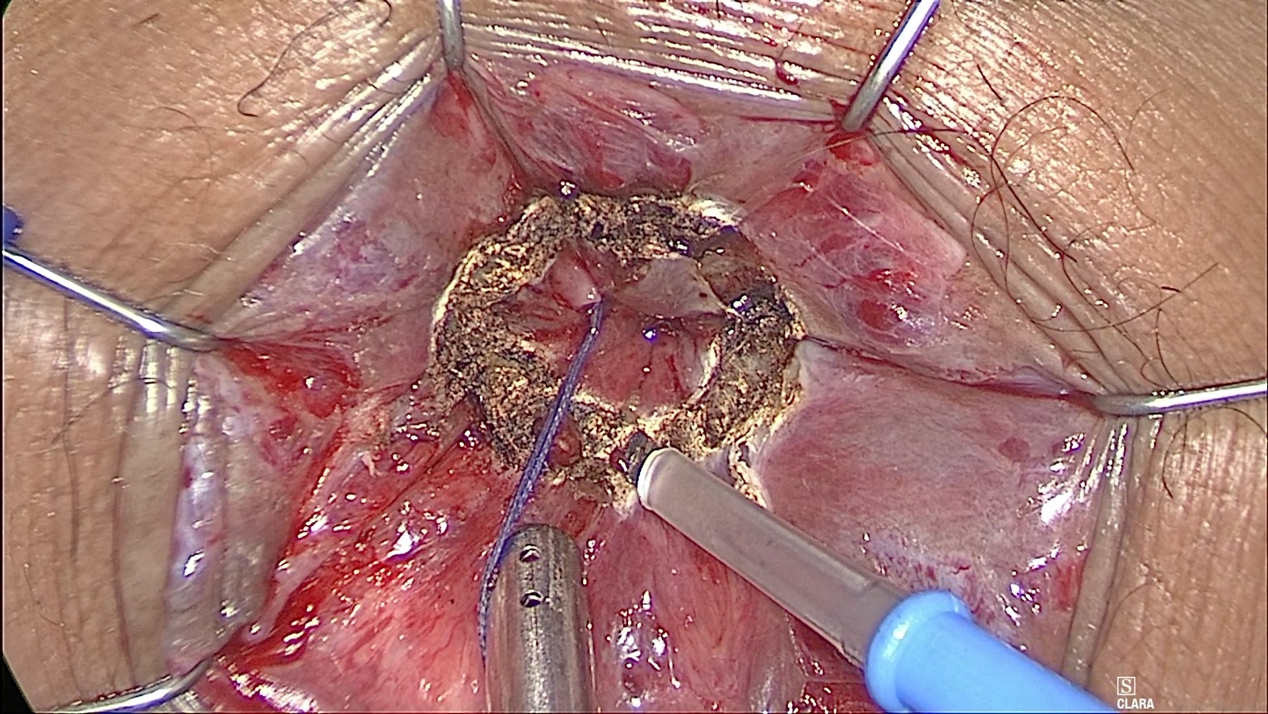


1. full-thickness dissection of the rectal wall and perirectal fat until the mesorectal plane was identified


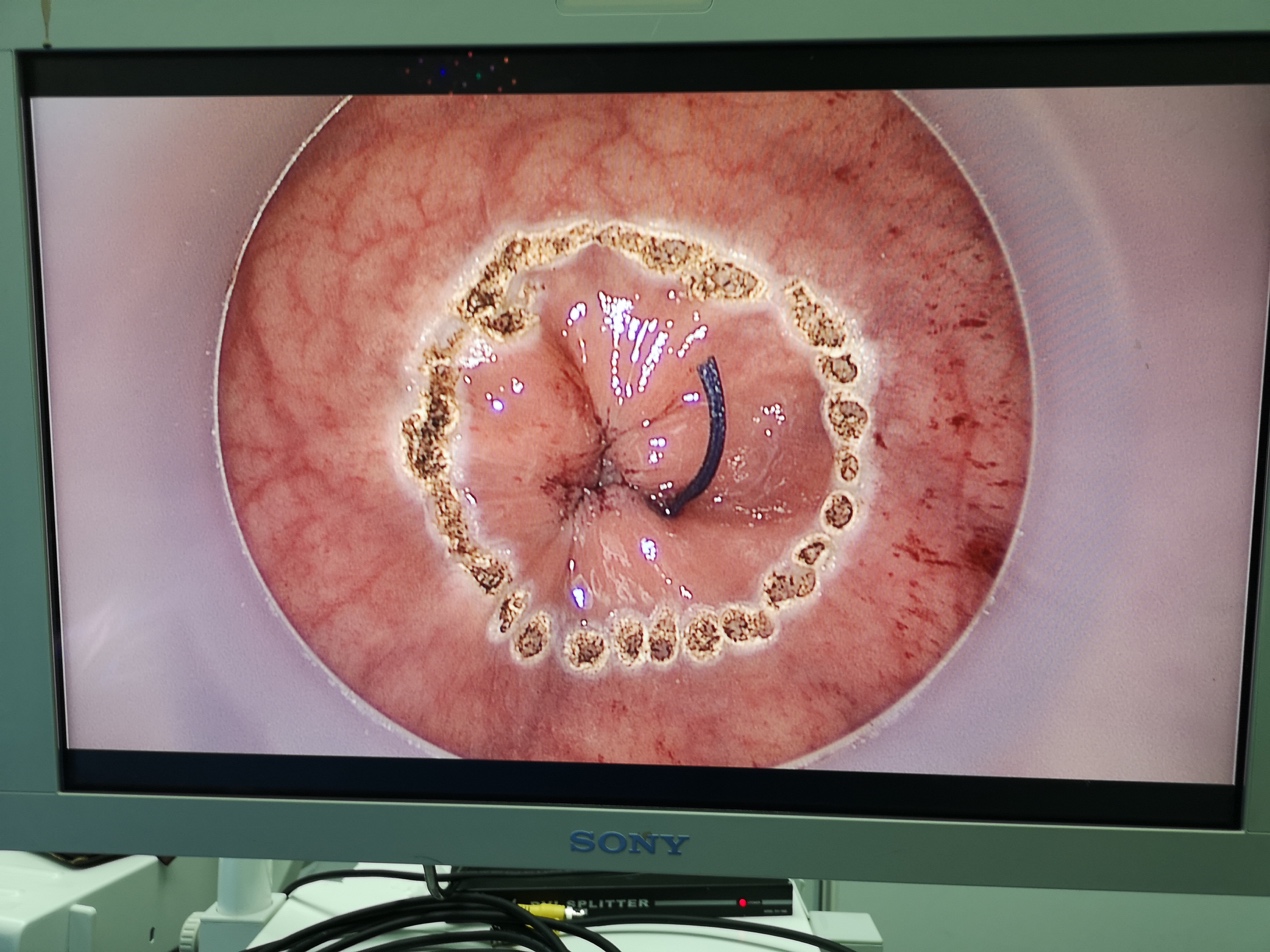


1. Setting-up of the transanal multi-channel working platform and establishing the pneumo-anorectum by insufflating CO2. (For a middle rectal tumor, step 3 was performed prior to steps 1 and 2)


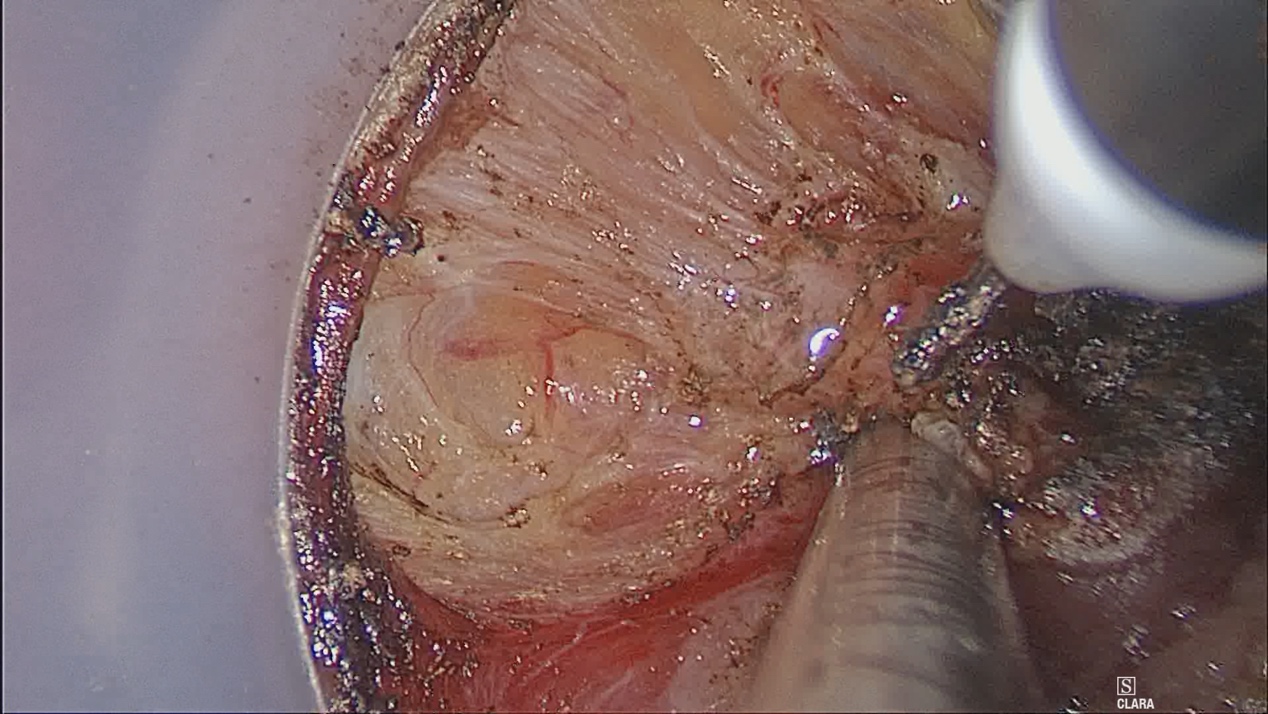


Lateral initiation of dissection of the entire intestinal wall


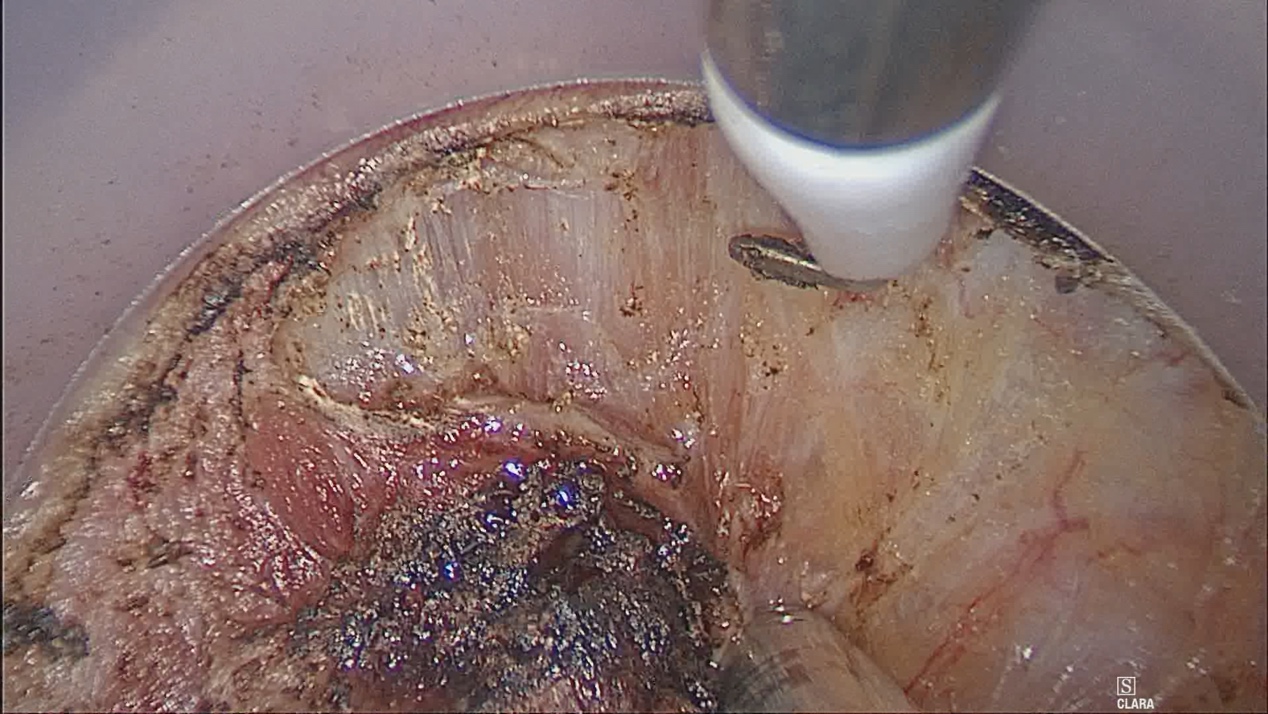


Anterior dissection of the united longitudinal muscles to approach the correct plane


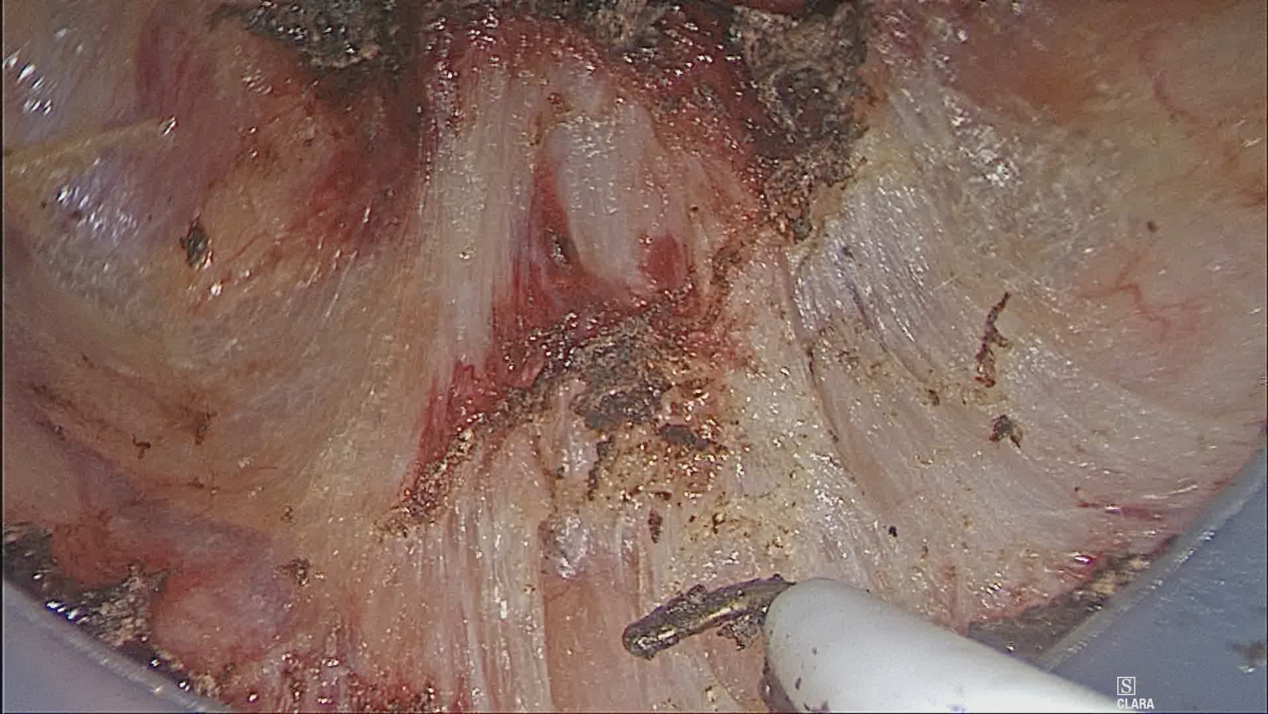


Posterior incision of the Hiatal ligament


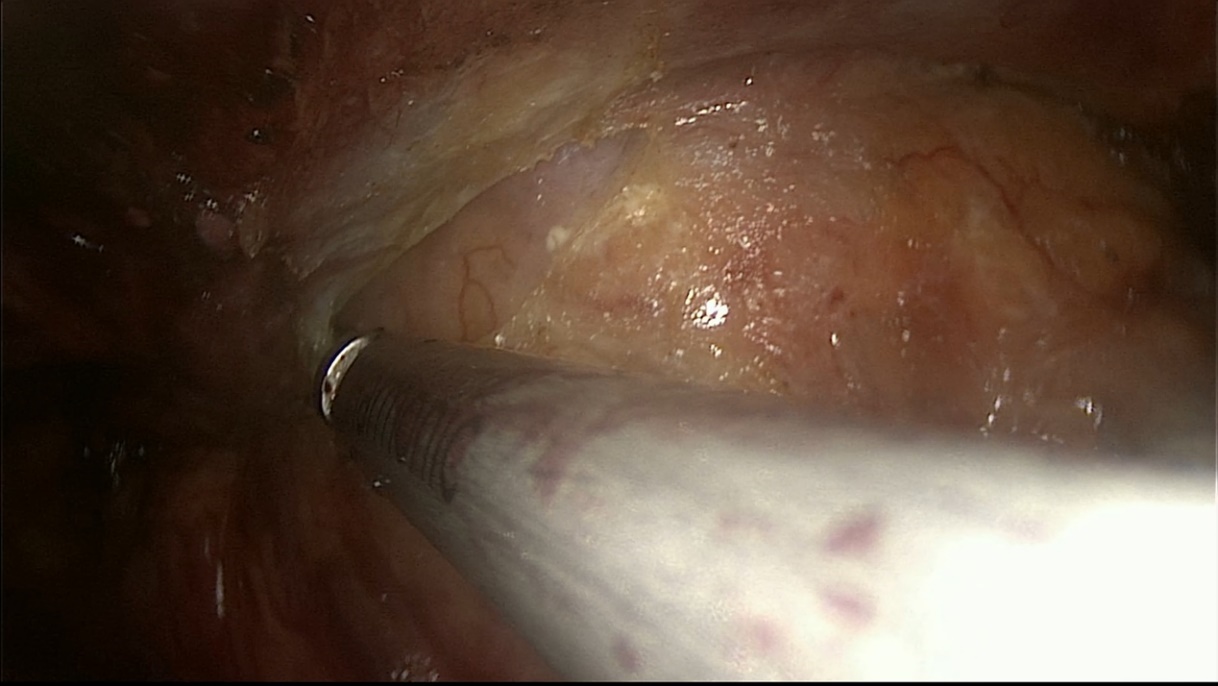


Incision peritoneal reflection


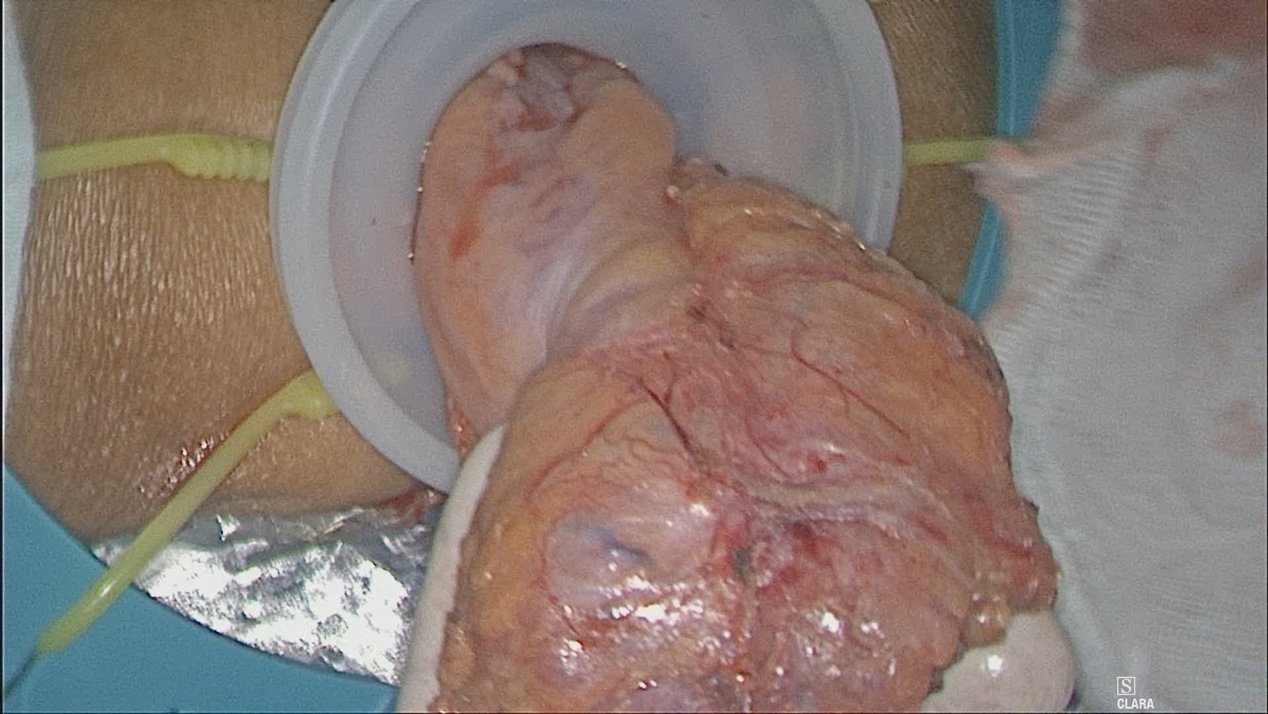


Delivering the specimen through anal


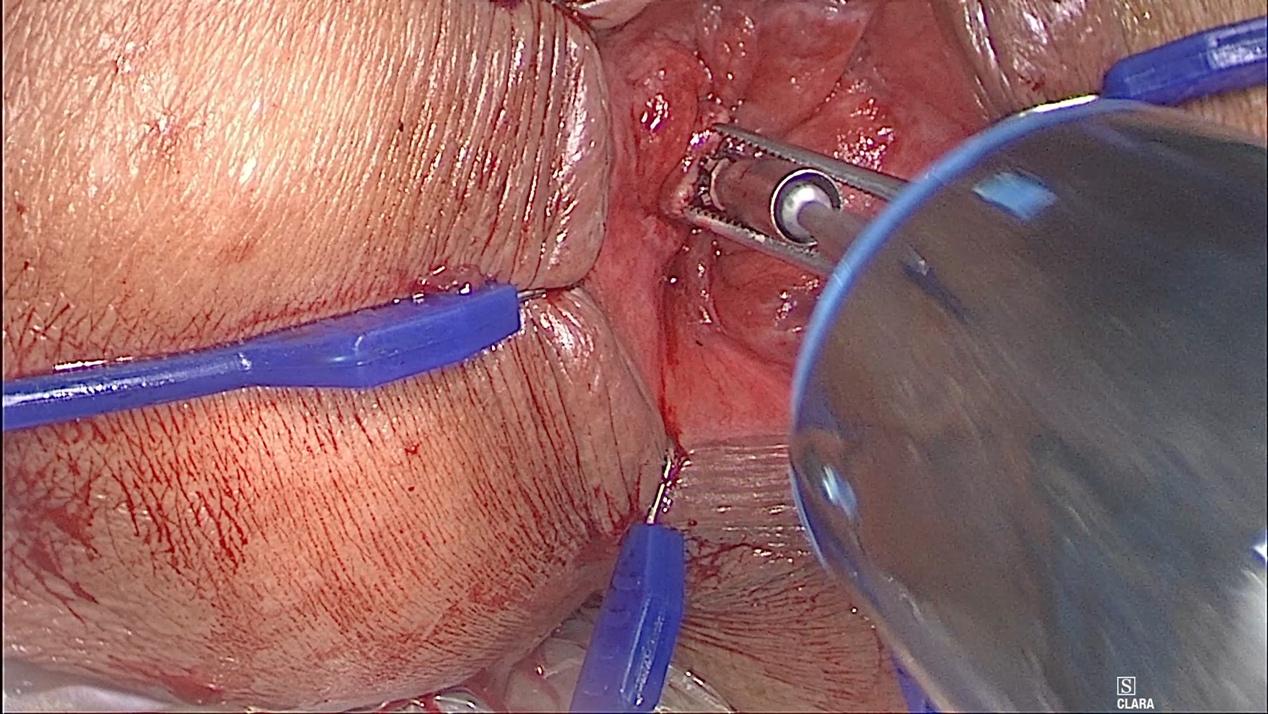


End-to-end straight stapled anastomosis


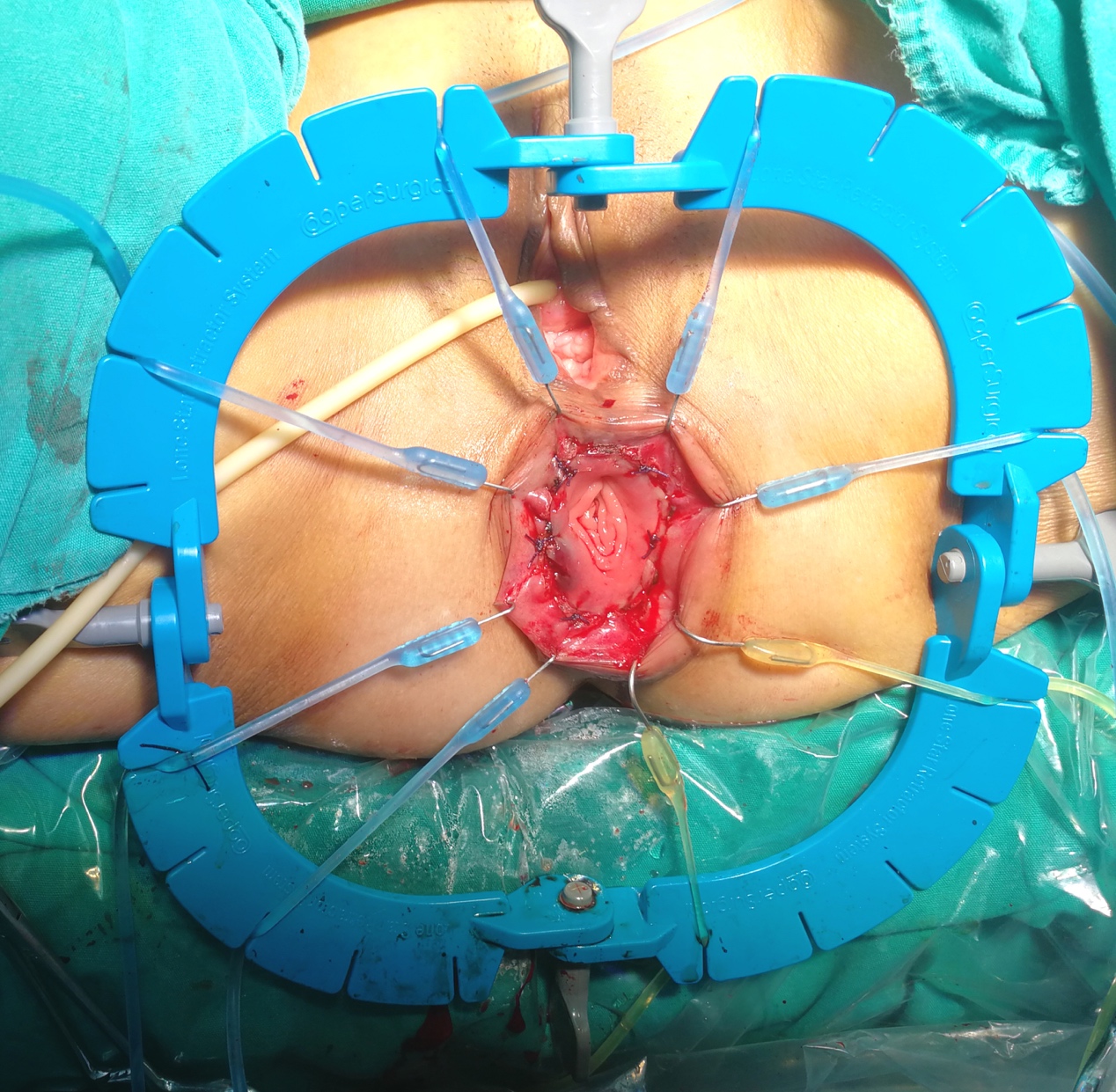


2-0 Vicryl handsewn anastomosis

**References**

[1] Kang L, Chen WH, Luo SL, Luo YX, Liu ZH, Huang MJ, et al. Transanal total mesorectal excision for rectal cancer: a preliminary report. Surg Endosc. 2016;30(6):2552–62.

[2] Kang L, Chen YG, Zhang H, Zhang HY, Lin G Le, Yang YC, et al. Transanal total mesorectal excision for rectal cancer: A multicentric cohort study. Gastroenterol Rep. 2020 Feb 1;8(1):36–41.

[3] Zeng Z, Luo S, Chen J, Cai Y, Zhang X, Kang L. Comparison of pathological outcomes after transanal versus laparoscopic total mesorectal excision: a prospective study using data from randomized control trial. Surg Endosc. 2020 Sep 1;34(9):3956–62.
